# Corneal topographic indices of scheimpflug camera in type 2 diabetic and non-diabetic elderly populations

**DOI:** 10.1186/s12886-023-03169-9

**Published:** 2023-10-23

**Authors:** Alireza Hashemi, Mehdi Khabazkhoob, Foroozan Narooei, Abolghasem Mortazavi, Hassan Hashemi

**Affiliations:** 1 Noor Ophthalmology Research Center, Noor Eye Hospital, Tehran, Iran; 2 Department of Basic Sciences, School of Nursing and Midwifery, Shahid Beheshti University of Medical Sciences, Tehran, Iran; 3 Noor Research Center for Ophthalmic Epidemiology, Noor Eye Hospital, Tehran, Iran; 4 Department of Neurosurgery, Sina Hospital, Tehran University of Medical Sciences, Tehran, Iran

**Keywords:** Corneal topography, Pentacam, Diabetes Mellitus, Diabetic retinopathy

## Abstract

**Purpose:**

The present study was conducted to determine the corneal topographic indices of Scheimpflug camera in type 2 diabetes patients without diabetic retinopathy (DR), DR and non-diabetic elderly populations.

**Methods:**

A total of 1105 participants were selected using random cluster sampling from Tehran, Iran and categorized into three groups including type 2 diabetes patients without diabetic retinopathy (DR), DR and non-diabetic. The diabetic group had HbA1c levels ≥ 6.4% with no other systemic problems. The non-diabetic participants had normal eye findings and no systemic diseases. The pachymetric progression index (PPI) values were measured using the Pentacam AXL.

**Results:**

A total of 1105 participants including 429 diabetes patients (38.46% male) and 676 non-diabetic (38.76% male) subjects entered the study. Only PPIavg and PPImax were higher in the diabetics versus non-diabetics (P = 0.019 and 0.010, respectively). There was a significant difference in PPImax between the three groups (P = 0.036). There were significant differences only in index of vertical asymmetry (IVA), central keratoconus index (CKI), PPI average, and PPI max between different stages of diabetic retinopathy (DR) (P = 0.045, 0.005, 0.002, and 0.004, respectively). There was a significant difference in index of Surface Variance (ISV), index of vertical asymmetry (IVA), PPIavg, and PPImax between diabetes patients with and without DR (P = 0.016, 0.022, < 0.001, and < 0.001, respectively).

**Conclusion:**

According to the results, diabetes and DR change several topographic indices. In addition, the HbA1c level may affect pachymetric progression index max. Therefore, special attention should be paid to these patients for different treatment strategies.

## Introduction

The prevalence of type 2 diabetes mellitus (DM) is increasing due to the population ageing, lifestyle changes, and urbanization [1]. The chronic hyperglycemic state of diabetes can damage many body organs such as the eyes, nerves, kidneys, heart, and blood vessels over time [2]. Although diabetic retinopathy (DR) is the most serious ocular complication of diabetes and one of the causes of secondary blindness during adulthood, [3] studies have shown that this disease also leads to changes in the anterior eye segment, especially the cornea [4, 5].

Ophthalmologists use topographic indices to diagnose corneal ectasia, follow up the patients, and carry out treatment plans [6]. Since these patients are poor candidates for ocular surgery, it is necessary to be aware of the corneal changes induced by DM for treatment strategies and refractive surgeries. In addition, comorbidities cause poor refractive results and postoperative complications [7].

Few studies have evaluated corneal topographic indices induced by hyperglycemic changes, and the results of these studies are contradictory. Some studies found no difference in keratometric readings between diabetic and non-diabetic groups [8, 9]. A small number of studies reported that the corneal curvature radius changes in diabetic people [10]. According to other studies, diabetic patients have higher keratometry values while corneal topography indices do not differ between diabetics and non-diabetics [11–13].

In addition to the contradictory results and the small number of studies that have examined the topographic indices of type 2 diabetic patients, these studies suffer from other limitations such as the small sample size, inattention to the presence of DR, and lack of diabetic control. Therefore, it is necessary to conduct population-based studies to compare the corneal topographic indices between diabetic and non-diabetic elderly subjects considering DR and diabetic control.

A population-based study was conducted to compare corneal topographic indices between type 2 diabetic and non-diabetic elderly subjects. Furthermore, the corneal indices were compared between different groups based on DR and HbA1c level. The relationship between the parameters and HbA1c and FBS levels was also evaluated.

## Methods

A cross-sectional population-based study was performed on subjects aged 60 years and over in Noor Eye Hospital, Tehran, Iran, 2018–2019. Multistage cluster sampling was used to select the participants from 22 districts of Tehran. The participants were categorized into three groups including diabetics (type 2 diabetes patients (with and without diabetic retinopathy) with a HbA1c level ≥ 6.4%) and non-diabetics. The patients were excluded from the study if they had a history of other systemic diseases affecting the eye, used topical or systemic drugs affecting the eye, wore contact lenses, had a history of refractive surgery, or suffered from chronic, inflammatory, and infectious ocular diseases. The third group included non-diabetic subjects with normal eye findings, HbA1c < 6.4%, and no systemic problems.

HbA1c is a glycated protein and a metabolic product of glucose binding to N-terminal valine residue in the β chain of hemoglobin. It shows the average blood glucose over the past three months. The normal range of HbA1c is between 4 and 6.4% [14]. A HbA1c level below 7% is generally accepted for the treatment of diabetes and indicates a lower risk of long-term microvascular and macrovascular complications of diabetes [15]. The subjects were divided into three groups based on their metabolic status, including group 1 (HbA1c < 6.4%, non-diabetic group), group 2 (HbA1c between 6.4% and 7%, type 2 diabetic patients with good metabolic control) and group 3 (HbA1c ≥ 7%, type 2 diabetic patients with poor metabolic control). In addition, the diabetics were also classified based on the presence of DR [15].

Both eyes were examined at the same time in both groups and one eye was randomly used for analysis. A complete medical and ocular history was taken. The posterior and anterior segments were examined using a Haag-Streit slit lamp and a + 90 lens by an experienced ophthalmologist to rule out any abnormalities in the ocular surface and posterior segment, respectively.

The patients underwent Scheimpflug imaging using the Pentacam AXL (Oculus, Wetzlar, Germany). The keratometry readings (K1, K2, Kmean and Kmax), Q-value, and the parameters of corneal variance such as the Index of Surface Variance (ISV), Index of Vertical Asymmetry (IVA), Central Keratoconus Index (CKI), Keratoconus Index (KI), Index of Height Asymmetry (IHA) and Index of Height Decentration (IHD), inferior-superior (I-S), Anterior Average Radius of Curvature (ARC), Posterior Average Radius of Curvature (PRC), and Pachymetric Progression Index (PPI) were measured. It should be mentioned that all examinations were performed in one day. The Pentacam measurements were performed between 10 a.m. and 2 p.m. to minimize diurnal variation.

The SPSS software version 23 (IBM Inc., Chicago, Illinois, USA) was used for statistical analyses. The results are expressed as the mean ± standard deviation (SD). The normality of the data was tested using the Kolmogorov-Smirnov test. The Mann-Whitney U test was applied to assess the difference between the two groups. Kruskal-Wallis test was used to compare the measurements between three groups based on HbA1c level. In addition, the Spearman test was used to determine the relationship between the parameters and HbA1c and FBS levels. P-values < 0.05 were considered significant.

### Ethical issues

Informed consent was obtained from all participants. The principles of the Helsinki Declaration were followed in all stages of the study. The protocol of the study was approved by the Ethics Committee of the National Institute for Medical Research Development (NIMAD) under the auspices of the Iranian Ministry of Health (ethics code: IR.NIMAD.REC.1397.292).

## Results

A total of 1105 participants (38.64% male) including 429 diabetic (38.46% male) and 676 non-diabetic (38.76% male) subjects entered the study. The demographic characteristics of the participants are summarized in Table 1. There was a significant difference in age between males and females in all participants, diabetics, and non-diabetics (P < 0.001). The results showed no significant difference in HbA1c and FBS levels between the male and female subjects in all participants and diabetics. In the non-diabetic group, HbA1c was 0.1% higher in females than in males (P = 0.03), but there was no significant difference in FBS level between them. According to the results, there was a significant difference in age, HbA1c and FBS between diabetic and non-diabetic groups (P = 0.001, < 0.001, and < 0.001, respectively).

**Table 1:** Demographic data of participants

		Diabetic group (n = 429)		Non-diabetic group (n = 676)		Total participants (n = 1105)	
F:M ratio		1.59		1.58		1.59	
		**Mean ± SD**	**Range**	**Mean ± SD**	**Range**	**Mean ± SD**	**Range**
Age (y.o)	**Male**	69.12 ± 6.88	60 to 94	67.51 ± 5.56	60 to 87	68.13 ± 6.15	60 to 94
	**Female**	66.48 ± 4.81	60 to 82	65.58 ± 4.86	60 to 87	65.93 ± 4.86	60 to 87
	**Total**	67.50 ± 5.83	60 to 94	66.33 ± 5.23	60 to 87	66.78 ± 5.50	60 to 94
HbA1c (%)	**Male**	8.17 ± 1.31	6.40 to 11.80	5.42 ± 0.37	4.00 to 5.90	6.49 ± 1.59	4.00 to 11.80
	**Female**	8.19 ± 1.36	6.50 to 11.90	5.50 ± 0.31	4.30 to 5.90	6.55 ± 1.58	4.30 to 11.90
	**Total**	8.18 ± 1.34	6.40 to 11.90	5.47 ± 0.34	4.00 to 5.90	6.52 ± 1.50	4.00 to 11.90
FBS (mg/dL)	**Male**	208.14 ± 82.42	60 to 435	96.53 ± 13.79	60 to 139	139.66 ± 75.45	60 to 435
	**Female**	197.85 ± 80.21	84 to 519	95.64 ± 12.56	58 to 140	135.44 ± 71.30	58 to 519
	**Total**	201.81 ± 81.13	60 to 519	95.98 ± 13.05	58 to 140	137.07 ± 72.92	58 to 519

n: number; y.o: years old; M: male; F: female; HbA1c: Hemoglobin A1c; FBS: fasting blood sugar; SD: standard deviation

As shown in Table 2, there was a significant difference in PPIavg and PPImax between the diabetic and non-diabetic groups (P = 0.019 and 0.010, respectively). All other parameters were similar between the two groups.

**Table 2:** Comparison of corneal topographic indices between diabetic and non-diabetic groups

	Diabetic group (n = 429)		Non-diabetic group (n = 676)		
	**Mean ± SD**	**Range**	**Mean ± SD**	**Range**	P-value*
K1 (D)	44.19 ± 1.66	38.70 to 52.40	44.19 ± 1.59	37.90 to 49.90	0.826
K2 (D)	45.03 ± 1.75	40.70 to 57.10	45.03 ± 1.70	39.20 to 51.10	0.808
Mean K (D)	44.61 ± 1.67	39.70 to 54.75	44.61 ± 1.61	38.55 to 50.50	0.848
Kmax (D)	45.99 ± 2.04	41.81 to 64.52	46.00 ± 1.94	40.38 to 54.69	0.843
Q-value	-0.43 ± 0.17	-1.62 to 0.16	-0.42 ± 0.89	-3.49 to 3.89	0.466
ISV	19.56 ± 10.87	5.00 to 113.00	18.61 ± 8.75	6.00 to 94.00	0.210
IVA (mm)	0.16 ± 0.10	0.03 to 1.19	0.15 ± 0.08	0.03 to 0.93	0.100
KI	1.02 ± 0.04	0.84 to 1.24	1.02 ± 0.03	0.90 to 1.14	0.405
CKI	1.00 ± 0.01	0.92 to 1.11	1.00 ± 0.01	0.92 to 1.05	0.613
IHA (µm)	6.05 ± 5.62	0.00 to 56.50	5.95 ± 6.09	0.00 to 85.00	0.709
IHD (µm)	0.02 ± 0.01	0.00 to 0.14	0.02 ± 0.01	0.00 to 0.13	0.353
PPImin	0.74 ± 0.24	0.20 to 2.73	0.72 ± 0.26	-2.46 to 3.60	0.186
PPIavg	1.03 ± 0.26	0.55 to 3.05	1.00 ± 0.25	0.00 to 3.90	**0.019**
PPImax	1.34 ± 0.38	0.70 to 4.51	1.31 ± 0.44	0.75 to 6.70	**0.010**
I-S value	-0.02 ± 0.97	-5.34 to 5.50	-0.04 ± 0.89	-3.49 to 3.89	0.924
ARC (mm)	7.60 ± 0.31	5.76 to 9.54	7.59 ± 0.28	6.72 to 9.09	0.930
PRC (mm)	6.15 ± 0.28	4.17 to 7.03	6.16 ± 0.26	4.86 to 6.99	0.606

K1: flat keratometry; K2: steep keratometry; Kmax: maximum keratometry; ISV: Index of surface variance; IVA: Index of vertical asymmetry; KI: Keratoconus index; CKI: Central keratoconus index; IHA: Index of height asymmetry; IHD: Index of height decentration; PPI: pachymetric progressive index; Min: minimum; Max: maximum; Avg: average; I-S: inferior-superior; ARC: anterior average radius of curvature; PRC: posterior average radius of curvature; SD: standard deviation; D: diopter

* Mann-Whitney Test. P < 0.05 is statistically significant. Bold values are statistically significant

Based on the HbA1c level, the number of subjects in the non-diabetic group, diabetes patients with good metabolic control and diabetes patients with poor metabolic control, was 676 (61.18%), 115 (10.40%), and 314 (28.42%), respectively. The HbA1c level was 5.47 ± 0.34 (4–5.9%), 6.70 ± 0.17 (6.40–7%), and 8.72 ± 1.16 (7.10–11.90%) in the first, second, and third groups, respectively. Although there was a difference in PPI max between the three groups (P = 0.036), no significant difference was found in other parameters between the three groups (Table 3).

**Table 3:** Comparison of corneal topographic indices between participants based on HbA1c level

	Group 1 (n = 676)	Group 2 (n = 115)	Group 3 (n = 314)	
	**Mean ± SD**	**Mean ± SD**	**Mean ± SD**	P-value*
	5.47 ± 0.34	6.70 ± 0.17	8.72 ± 1.16	
K1 (D)	44.19 ± 1.59	44.24 ± 1.95	44.18 ± 1.54	0.869
K2 (D)	45.03 ± 1.70	45.14 ± 2.21	45.00 ± 1.55	0.901
Mean K (D)	44.60 ± 1.61	44.69 ± 2.06	44.59 ± 1.51	0.875
Kmax (D)	46.00 ± 1.94	46.14 ± 2.76	45.94 ± 1.70	0.961
Q-value	-0.42 ± 0.15	-0.43 ± 0.19	-0.43 ± 0.17	0.744
ISV	18.61 ± 8.75	19.81 ± 11.95	19.47 ± 10.48	0.412
IVA (mm)	0.15 ± 0.08	0.16 ± 0.12	0.16 ± 0.10	0.237
KI	1.02 ± 0.03	1.02 ± 0.04	1.02 ± 0.03	0.577
CKI	1.00 ± 0.01	1.00 ± 0.01	1.00 ± 0.01	0.629
IHA (µm)	5.95 ± 6.09	6.15 ± 5.45	6.02 ± 5.69	0.932
IHD (µm)	0.02 ± 0.01	0.02 ± 0.02	0.02 ± 0.01	0.631
PPImin	0.72 ± 0.26	0.74 ± 0.25	0.74 ± 0.23	0.276
PPIavg	1.00 ± 0.25	1.05 ± 0.30	1.02 ± 0.24	0.062
PPImax	1.30 ± 0.44	1.36 ± 0.47	1.33 ± 0.35	**0.036**
I-S value	-0.04 ± 0.89	0.05 ± 1.16	-0.05 ± 0.90	0.609
ARC (mm)	7.59 ± 0.28	7.58 ± 0.37	7.60 ± 0.29	0.982
PRC (mm)	6.16 ± 0.26	6.14 ± 0.35	6.15 ± 0.25	0.812

K1: flat keratometry; K2: steep keratometry; Kmax: maximum keratometry; ISV: Index of surface variance; IVA: Index of vertical asymmetry; KI: Keratoconus index; CKI: Central keratoconus index; IHA: Index of height asymmetry; IHD: Index of height decentration; PPI: pachymetric progressive index; Min: minimum; Max: maximum; Avg: average; I-S: inferior-superior; ARC: anterior average radius of curvature; PRC: posterior average radius of curvature; SD: standard deviation; HbA1c: glycosylated hemoglobin; Group 1: HbA1c ˂6.4%, Group 2: HbA1c up to 7%, Group 3: HbA1c ˃7% * Kruskal-Wallis test. P < 0.05 is statistically significant. Bold value is statistically significant

According to Table 4, FBS had a significant correlation with K1, K2, mean K, Kmax, and ARC with in non-diabetic group (P = 0.010, 0.017, 0.009, 0.024, and 0.037, r = 0.099, 0.103, 0.100, 0.087, and − 0.080, respectively). Furthermore, FBS had a significant correlation with PPImin and PRC in the diabetic group (P = 0.022 and 0.018, r = 0.110 and − 0.114, respectively). Although there was a significant correlation between I-S value and HbA1c in the diabetic group (P = 0.016, r=-0.117), the results showed no significant correlation between the study parameters and HbA1c in the non-diabetic group.

**Table 4:** The correlation coefficients among anterior corneal topographic indices, HbA1c and fasting blood sugar levels in diabetic and non-diabetic groups

		FBS		HbA1c	
		P-value*	**r**	P-value*	**r**
K1 (D)	**Diabetic group**	0.238	0.057	0.820	0.011
	**Non-diabetic group**	**0.010**	0.099	0.918	0.004
K2 (D)	**Diabetic group**	0.119	0.075	0.708	0.018
	**Non-diabetic group**	**0.007**	0.103	0.373	0.034
Mean K (D)	**Diabetic group**	0.148	0.070	0.744	0.016
	**Non-diabetic group**	**0.009**	0.100	0.560	0.022
Kmax (D)	**Diabetic group**	0.498	0.033	0.992	0.000
	**Non-diabetic group**	**0.024**	0.087	0.362	0.035
Q-value	**Diabetic group**	0.720	0.017	0.403	-0.040
	**Non-diabetic group**	0.479	0.027	0.912	-0.004
ISV	**Diabetic group**	0.541	-0.030	0.731	0.017
	**Non-diabetic group**	0.546	-0.023	0.096	0.064
IVA (mm)	**Diabetic group**	0.122	-0.075	0.749	-0.016
	**Non-diabetic group**	0.308	-0.039	0.735	0.013
KI	**Diabetic group**	0.390	-0.042	0.159	-0.068
	**Non-diabetic group**	0.859	0.007	0.772	0.011
CKI	**Diabetic group**	0.130	-0.073	0.823	-0.011
	**Non-diabetic group**	0.753	-0.012	0.589	-0.021
IHA (µm)	**Diabetic group**	0.107	-0.078	0.674	-0.020
	**Non-diabetic group**	0.449	-0.029	0.534	-0.024
IHD (µm)	**Diabetic group**	0.524	-0.031	0.884	-0.007
	**Non-diabetic group**	0.424	0.031	0.682	-0.016
PPImin	**Diabetic group**	**0.022**	0.110	0.672	0.021
	**Non-diabetic group**	0.244	-0.045	0.761	-0.012
PPIavg	**Diabetic group**	0.243	0.056	0.784	-0.013
	**Non-diabetic group**	0.135	-0.058	0.996	0.000
PPImax	**Diabetic group**	0.469	0.035	0.605	-0.025
	**Non-diabetic group**	0.462	-0.028	0.690	0.015
I-S value	**Diabetic group**	0.088	-0.082	**0.016**	-0.117
	**Non-diabetic group**	0.636	0.018	0.790	0.010
ARC (mm)	**Diabetic group**	0.082	-0.084	0.859	-0.009
	**Non-diabetic group**	**0.037**	-0.080	0.392	-0.033
PRC (mm)	**Diabetic group**	**0.018**	-0.114	0.933	-0.004
	**Non-diabetic group**	0.544	-0.023	0.425	-0.031

K1: flat keratometry; K2: steep keratometry; Kmax: maximum keratometry; ISV: Index of surface variance; IVA: Index of vertical asymmetry; KI: Keratoconus index; CKI: Central keratoconus index; IHA: Index of height asymmetry; IHD: Index of height decentration; PPI: pachymetric progressive index; Min: minimum; Max: maximum; Avg: average; I-S: inferior-superior; ARC: anterior average radius of curvature; PRC: posterior average radius of curvature; HbA1c: glycosylated hemoglobin; FBS: fasting blood sugar.* The Spearman’s Rank Correlation Test. P < 0.05 is statistically significant. Bold values are statistically significant

Of 429 diabetic subjects, 366 (85.31%) did not have DR while 47 (10.95%), 6 (1.40%), 5 (1.17%), and 5 (1.17%) had mild non-proliferative diabetic retinopathy (NPDR), moderate NPDR, severe NPDR, and PDR, respectively. Kruskal-Wallis Test showed significant differences only in IVA, CKI, PPIAvg, and PPImax among different stages of DR (P = 0.045, 0.005, 0.002, and 0.004, respectively). In addition, the diabetic subjects were grouped into two groups based on the presence of DR. According to the results, there was a statistically significant difference in ISV, IVA, PPIavg, and PPImax between diabetic subjects with and without DR (P = 0.016, 0.022, < 0.001, and < 0.001, respectively). All other parameters were similar between the two groups. (Table 5)

**Table 5:** Comparison of corneal topographic indices between diabetics with and without DR

	Diabetic group without DR (n = 366)	Non-diabetic group with DR (n = 63)	
	**Mean ± SD**	**Mean ± SD**	P-value*
K1 (D)	44.14 ± 1.63	44.47 ± 1.79	0.217
K2 (D)	44.99 ± 1.72	45.25 ± 1.89	0.465
Mean K (D)	44.57 ± 1.64	44.86 ± 1.82	0.323
Kmax (D)	46.00 ± 2.03	45.92 ± 2.06	0.606
Q-value	-0.43 ± 0.18	-0.42 ± 0.15	0.857
ISV	19.94 ± 11.23	17.35 ± 8.24	**0.016**
IVA (mm)	0.16 ± 0.11	0.13 ± 0.06	**0.022**
KI	1.02 ± 0.04	1.02 ± 0.02	0.786
CKI	1.00 ± 0.01	1.00 ± 0.01	0.121
IHA (µm)	6.23 ± 5.85	5.00 ± 3.92	0.262
IHD (µm)	0.02 ± 0.02	0.01 ± 0.01	0.053
PPImin	0.74 ± 0.25	0.75 ± 0.15	0.145
PPIavg	1.02 ± 0.27	1.08 ± 0.15	**< 0.001**
PPImax	1.33 ± 0.40	1.41 ± 0.22	**< 0.001**
I-S value	-0.04 ± 1.01	0.07 ± 0.74	0.262
ARC (mm)	7.60 ± 0.31	7.54 ± 0.29	0.187
PRC (mm)	6.16 ± 0.28	6.09 ± 0.28	0.266

K1: flat keratometry; K2: steep keratometry; Kmax: maximum keratometry; ISV: Index of surface variance; IVA: Index of vertical asymmetry; KI: Keratoconus index; CKI: Central keratoconus index; IHA: Index of height asymmetry; IHD: Index of height decentration; PPI: pachymetric progressive index; Min: minimum; Max: maximum; Avg: average; I-S: inferior-superior; ARC: anterior average radius of curvature; PRC: posterior average radius of curvature; SD: standard deviation; * Mann-Whitney Test. P < 0.05 is statistically significant. Bold value is statistically significant

## Discussion

The patients with diabetes mellitus (DM) can be poor candidates for refractive surgery. However, despite the widespread use of corneal topography and tomography, the possible effect of DM on corneal topographic changes has not been well studied. In the present study, corneal topography indices were compared between diabetic and non-diabetic subjects as well as diabetic subjects with and without DR. The results of corneal topography indices in the elderly population aged 60 years and over with and without DM provide a suitable reference for comparison with other studies with similar age ranges. In addition, knowledge of the difference in the mentioned parameters between diabetics with and without DR can complete the results of other studies.

Comparisons between the diabetic and non-diabetic groups showed that only PPIavg and PPImax were statistically higher in the diabetic versus non-diabetic subjects. Tomography-derived pachymetric indices such as PPI values are better for the differential diagnosis of normal from abnormal corneas compared to single-point corneal thickness measurements [16, 17]. Several studies compared central corneal thickness between the diabetic and non-diabetic groups [18]. Although a number of studies found that diabetic patients had thicker corneas compared to non-diabetic subjects, [19, 20] some other studies did not report any difference between the two groups [5, 19].

According to many studies, DM causes abnormal changes in corneal morphology and function of endothelial cells. Functional disturbances may increase the autofluorescence of the cornea and its penetrability [21]. Since glucose enters the eye through the retinal and choroidal blood circulation following hyperglycemia, it reaches the crystalline lens and cornea via the vitreous and aqueous humor. Therefore, hyperglycemia can also affect the corneal topography via changing corneal hydration [22, 23].

In contrast to the present study, Huseynova et al. [11] found that Kmax was higher in the diabetic versus the non-diabetic group. However, corneal topography indices did not differ between the two groups. Diabetic retinopathy was not observed in any of the 18 diabetic subjects.

In another study, the corneal topographic and keratometric changes in diabetic patients who were under intensive treatment for acute severe hyperglycemia were evaluated using C-scan corneal topography and Javal keratometer, respectively [24]. A significant change was found in the keratometric measurements at the flattest corneal meridian, but no statistically significant changes were found in Javal keratometric data after intensive treatment. None of the participants had signs of PDR.

Similar to the present results, several studies reported that HbA1c levels did not affect corneal curvature and asphericity [24, 25].

In the present study, a limited number of diabetic subjects had progressive stages of DR, which may be due to excluding the diabetic subjects with other systemic problems.

One of the reasons for the difference between the studies is the measurement method. Another reason could be differences in the study design and mixing type 1 and type 2 DM subjects. Differences in the sample size, measurement method, age range, mixing type 1 and type 2 diabetic subjects, and inattention to the associated DR in previous studies can be considered as possible reasons for the difference between the results of the present study and other studies.

One of the strengths of the present study was evaluation of corneal topography indices of the elderly population considering DR status and stage and the HbA1c level. However, this study had some limitations. First, it had a cross-sectional design, which makes it impossible to evaluate the causal relationship between diabetes and corneal topography indices. Second, we did not evaluate the corneal endothelium. Therefore, it is recommended that future longitudinal studies be performed to evaluate status of the corneal endothelium.

In summary, several corneal topographic parameters change following hyperglycemia and the presence of DR. Therefore, it is recommended to carefully measure corneal topography indices of diabetic subjects before refractive surgery.

## Data Availability

The datasets used and/or analyzed during the current study available from the corresponding author on reasonable request.
